# Social Inequalities in Use of Preventive Dental and Medical Services among Adults in European Countries

**DOI:** 10.3390/ijerph16234642

**Published:** 2019-11-22

**Authors:** Shiho Kino, Eduardo Bernabé, Wael Sabbah

**Affiliations:** 1 Department of Social and Behavioral Sciences, Harvard T.H. Chan School of Public Health, University of Boston, Boston, MA 02115, USA; shkino@hsph.harvard.edu; 2 Faculty of Dentistry, Oral & Craniofacial Sciences, King’s College London, London SE5 9RS, UK; wael.sabbah@kcl.ac.uk

**Keywords:** socioeconomic factors, social determinants of health, healthcare disparities, preventive health services, Europe, adult

## Abstract

This study examined inequalities in dental check-ups and medical screenings using subjective and objective socioeconomic indicators. Data from 23,464 adults, aged 20 years old and over, who participated in a multi-national survey across Europe (Eurobarometer 72.3) were analysed. Participants’ socioeconomic position (SEP) was measured by education, difficulty in paying bills and subjective social status. Use of preventive services was measured by attendance for dental check-ups, cancer and cardiovascular screenings in the past 12 months. Socioeconomic inequalities were assessed in two-level logistic regression (adults nested within countries), adjusting for demographic factors and type of healthcare system. There were apparent social inequalities in using all three preventive services. However, only dental check-ups showed consistent and significant inequalities across all socioeconomic indicators with those in the bottom groups in education (odds ratio: 0.51; 95% confidence interval: 0.46–0.55), difficulty in paying bills (OR: 0.64; 95% CI: 0.59–0.72) and subjective social status (OR: 0.63; 95% CI: 0.57–0.69) having lower odds of reporting dental check-ups in the past 12 months than those in the top groups. Cancer screening was not associated with difficulty in paying bills whereas cardiovascular disease screening was not associated with education and subjective social status. Despite the availability of universal health coverage, there were clear social gradients in using preventive services particularly across education and subjective social status groups. The stronger and more consistent gradients observed in dental check-ups compared to cancer and cardiovascular screening could be attributed to difference in the level of coverage of dental and medical services in Europe.

## 1. Introduction

Screenings and check-ups for medical and dental conditions have been identified as important contributors to reduction in the burden of diseases as they help to identify high risk individuals and enable timely intervention [1,2]. Cancer screening is associated with lower rates of cancer-related mortality [3,4]. Similarly, cardiovascular screening is linked to lower rates of cardiovascular disease-related mortality [5]. Regular dental attendance for check-ups are also related to lower rates of dental caries, periodontal disease and tooth loss [6,7].

The use of healthcare services is influenced by a number of individual and contextual factors [8]. Social inequalities in use of preventive healthcare services are likely to exacerbate the existing inequalities in health [9], particularly in countries where the healthcare system does not provide universal medical coverage [10,11]. In Europe, almost all member countries of the European Union (EU) have a healthcare system that provides universal coverage for medical conditions [12], which usually includes tests and screening for life-threatening conditions, such as cancer and cardiovascular disease [12]. On the contrary to medical care, oral healthcare is not universally covered in many European countries [13]. For example, in several EU countries, adults can obtain treatment subsidised either from general taxation or national insurance systems [13]. On the other hand, a smaller proportion of the adult populations in Southern and Eastern Europe are fully covered by either public or private dental insurance [13], thus increasing the financial burden of access to, and use of dental services. Given the variations in the levels of coverage across EU countries, it is reasonable to hypothesise that the use of preventive dental services is more unequal than the use of preventive medical services.

Most studies assessing inequalities in the use of healthcare services used objective indicators of socioeconomic position such as income and education [14]. While subjective social status does not necessarily reflect material ability, it might be a more consistent indicator when used across countries [15]. This study examined inequalities in use of preventive dental and medical services, according to subjective and objective socioeconomic indicators, among adults in 27 European countries.

## 2. Materials and Methods

### 2.1. Study Population

Data were from the Eurobarometer 72.3: Public Health Attitude, Behaviour, and Prevention. This was a cross-sectional survey conducted in 2009, which included 30,292 participants, aged 15 years old and over, residing in the 27 EU countries and 3 candidate countries. The survey is available at https://www.icpsr.umich.edu/icpsrweb/ICPSR/studies/32441. In every participating country, a multi-stage random sample was drawn with the aim to recruit 1000 people. The United Kingdom (Great Britain = 1000, Northern Ireland = 300) and Germany (East = 500, West = 1000) had larger samples whereas Cyprus Republic, Luxembourg, Malta and Turkey had smaller samples (500 each). Data were collected through home interviews. Only one interview was carried out per household [16]. Respondents in every country provided written informed consent before participation.

There were 25,350 adult participants, aged 20 years old and older, in the 27 EU countries. The survey questionnaire in the three candidate countries did not include the section on use of preventive services and thus, participants in those countries were excluded. The lower limit for age was set at 20 years old based on international recommendations to screen for specific types of cancers (cervical) [17,18] and cardiovascular risk factors (blood pressure and cholesterol) [19,20] from such an age. Therefore, the study sample included 23,464 (92.6%) participants after excluding those with missing values.

### 2.2. Selection of Variables

The outcome measure was the use of preventive dental and medical services, determined by attendance for dental check-ups and screenings for cancer and cardiovascular disease (CVD) in the past 12 months, respectively. The original question was “For each item, please tell me if you had one in the last 12 months, whether or not as part of any treatment”. There were 10 items: dental check-up; X-ray, ultrasound or other scan; eye test by an optician or an eye doctor; cholesterol test; heart check-up; hearing test; blood pressure test; colorectal cancer testing (faecal occult blood test); prostate specific antigen test; and other test for cancer. Response options for each item were yes, on my own initiative (1); yes, on doctors initiative (2); yes, in a screening programme (3); and no (4). Dental check-ups were indicated by a positive response (codes 1 to 3). Cancer screening was indicated by a positive response (codes 1 to 3) for either colorectal, prostate or other cancer tests. CVD screening was a positive response (codes 1 to 3) for either cholesterol test, heart check-up or blood pressure examination.

The exposure of interest was socioeconomic position (SEP) which was indicated by education, difficulty in paying bills and subjective social status. Education is a more comparable SEP indicator across different countries [21]. Difficulty in meeting bills indicates financial ability and income conditions [22,23]. Subjective social status is a measure of social standing useful for multi-nation comparisons because it indicates an individual’s perception of their status in their society [24,25]. Education was indicated by the age at which full-time education was completed. This variable was recorded as 20 years old or more, 16–19 years old and below 15 years old [26,27]. Difficulty in paying bills was reported as cannot pay bills most of the time, from time to time and almost never/never. For subjective social status, participants were asked to place themselves on a ladder (scale from 1 to 10) indicating their perception of own position in their respective society. As in previous studies responses were categorised into four groups [25,26]: highest (7 to 10), second highest (6), second lowest (5) and the lowest (1 to 4). All SEP indicators were recoded so that the reference group had the best conditions (most educated, least difficulty in paying bills and highest subjective social status).

Several individual- and country-level factors were selected as confounders based on Andersen’s behavioural model of health services use [8]. Gender, age, area of residence and marital status were the individual-level confounders. The type of healthcare system was the only country-level confounder. Countries were classified into 5 groups based on their administrative system and finance of healthcare, namely Nordic (Denmark, Finland and Sweden), Bismarckian (Austria, Belgium, France, Germany, Luxembourg and the Netherlands), Beveridgean (United Kingdom and Ireland), Southern European (Greece, Italy, Malta, Portugal, Spain and Cyprus) and Eastern European (Bulgaria, Czech Republic, Hungary, Poland, Romania, Slovakia, Estonia, Latvia, Lithuania and Slovenia) [13].

### 2.3. Statistical Analysis

Analytical weights were used for the descriptive analysis to produce nationally representative estimates. The proportion of adult participants using preventive dental and medical services was compared by confounders using the Chi-squared test. Linear trends in the use of preventive services according to each SEP indicator were tested by fitting the SEP indicator as a continuous variable in the logistic regression model. These analyses were done in Stata SE 15 (StataCorp., TX, United States).

Socioeconomic inequalities in the use of dental check-ups, cancer screening and CVD screening were explored in separate two-level binary logistic regression models, to account for the clustering of individuals within countries. We presented fully adjusted models for each outcome, including the three SEP indicators and all confounders at individual- (age, gender, area of residence and marital status) and country-level (healthcare system). Multilevel models were estimated in MLwiN 2.35 (Centre for Multilevel Modelling, University of Bristol, Bristol, England). We did not use sampling weights for multilevel analyses because the survey did not provide country-level weights.

Sensitivity analysis was conducted for those aged 40 years old or over and those aged 50 years old or over to confirm that the observed socioeconomic inequalities, particularly in CVD screening and cancer screening, if any, were independent from the age effect.

## 3. Results

Data from 23,464 (52.1% women) adults with complete data on relevant variables were analysed. Their mean age was 48.4 years old (SD: 17.3, range: 20–96). The study sample is described in Table 1. There were no major differences in the main outcomes and explanatory variables between those included and excluded from the analysis. In the past 12 months, 69.6% (95% CI: 68.9–70.6) of participants had CVD screening, 63.1% (95% CI: 62.1–64.0) had a dental check-up and 24.1% (95% CI: 23.2–24.9) had cancer screening. Table 2 shows socioeconomic inequalities in use of preventive services. Significant positive linear trends in use of dental check-ups, cancer screening and CVD screening were observed according to education, difficulty in paying bills and subjective social status.

Results from the multilevel logistic regression models are presented in Table 3. Only 5.5%, 3.5% and 5.7% of the total variance in use of dental check-ups, cancer screening and CVD screening were left unexplained at country-level, respectively. Socioeconomic inequality was consistently significant and demonstrated a social gradient in dental check-ups, while it was less consistent in cancer and CVD screening. Participants with the lowest education (OR: 0.51; 95% CI: 0.46–0.55), lowest subjective social status (OR: 0.63; 95% CI: 0.57–0.69) and having difficulty in paying bills most of the time (OR: 0.64; 95% CI: 0.58–0.72) had lower odds of attending a dental check-up in the past 12 months. Participants with the lowest education (OR: 0.68, 95% CI: 0.61–0.75) and lowest subjective social status (OR: 0.71, 95% CI: 0.63–0.79) had lower odds of attending cancer screening in the past 12 months. Finally, participants with the lowest subjective social status (OR: 0.78; 95% CI: 0.71–0.87) and having difficulty in paying bills most of the time (OR: 0.81; 95% CI: 0.73–0.91) had lower odds of attending CVD screening in the past 12 months. In sensitivity analyses for those aged 40 years old or over, and those aged 50 years old or over, similar social inequalities were observed in CVD and cancer screening and dental check-ups.

## 4. Discussion

This study examined socioeconomic inequality in using preventive dental and medical services, namely dental check-ups as well as cancer and CVD screening, among adults in the 27 European Union countries using objective and subjective SEP indicators. Attendance for all dental and medical services was significantly more common among participants in higher socioeconomic position. However, dental check-ups showed more consistent and significant inequalities across all SEP indicators than cancer and CVD screening. Furthermore, the random parts of the models indicated that the around 95% of the total variance for each of the outcomes were explained by individual-level factors rather than country-level factors. This observation demonstrates the detrimental role of individual-level rather than country-level factors in explaining variations in use of preventive services.

These findings in Europe appear to be similar to findings in Canada which showed income and education gradients in use of dental services and inversed gradients in use of medical services [28], although different SEP indicators were used in the two studies. The greater inequality in dental check-ups seen in this study could be attributed to the fact that CVD and cancer are covered by universal healthcare system in most European countries on contrary to dental care, which could be partially attributed to the perception of cardiovascular disease and cancer as being life-threatening conditions compared to oral diseases [13]. On the other hand, aside from the impact of severe deprivation indicated by difficulty in paying bills most of the time, CVD screening was relatively equitably distributed among education and subjective social status groups. The moderate socioeconomic inequality in CVD screening could also be attributed to the inclusion of routine procedures, such as blood pressure assessments.

The greater and consistent inequality observed in dental check-ups in comparison to cancer and CVD screenings appears to be logical, but not justifiable, given the availability of public coverage for these two conditions in most countries due to their perceived life-threatening nature [29]. The fact that socioeconomic inequality existed across education and subjective social status in cancer screening, but that inequality existed in CVD screening only across difficulty in paying bills could be because cancer screening is influenced by a person’s knowledge and it is more sensitive to psychological factors than CVD. In addition to the perception of the risk of acquiring cancer, it is also possible that doctors’ initiative for attending cardiovascular disease screening was higher than that for cancer screening [30]. Furthermore, screening for some specific cancers might be recommended at specific time intervals for certain ages. The question in the survey enquired only about attendance of cancer screening last year and could have missed those who were screened earlier than a year ago.

Almost all EU countries included in this analysis have universal medical coverage which covers direct costs for medical examinations [12]. This universal coverage could partially explain the smaller inequalities in cancer and CVD screening. However, the observation that inequalities still existed in different indicators for cancer and CVD screening also indicates that universal medical coverage does not eliminate inequality in the use of these services. It also underscores the indirect barriers (time lost in waiting and travel as well as productivity loss) faced by socially disadvantaged groups in society [31]. On the other hand, studies conducted in countries with no universal health services coverage, such as the United States, have shown greater inequality in cancer and cardiovascular screenings for early detection of the diseases [32,33]. Furthermore, the inequality in the United States also exists among under-served immigrant and ethnic minority communities, not just by income and education [34].

The findings of the observed high inequality in dental check-ups, and relatively moderate inequality in cancer and CVD screenings are likely to exacerbate the existing inequality in general and oral health and related mortality. Early detection of cancer, precancerous lesions and risk factors for CVD would at least save lives and reduce mortality [5]. Furthermore, it could also lead to more successful and less costly interventions. Reducing inequality in regular cancer screening should be a major priority for health policies given the usually poor prognosis and the high cost of care. Similarly, most oral diseases are avoidable with proper preventive behaviours and intervention. Dental check-ups play an important role in maintaining good oral health and is linked to a better quality of life [35]. The importance of prevention of these conditions should be emphasised to promote individuals’ and practitioners’ initiative towards early detection. Furthermore, new health policies that aim at eliminating indirect costs in the use of preventive services should be considered. Financial stresses related to difficulty in paying bills and psychological consequences of inequalities exacerbate the risk for cardiovascular diseases, cancer and oral diseases, particularly periodontitis, which puts the most disadvantaged individuals at an even greater risk of mortality and morbidity related to these conditions [34,35,36,37].

Some limitations of this study need to be addressed. Firstly, the use of cross-sectional data cannot establish SEP preceded use of services. Therefore, we cannot conclude that lack of use of preventive services is due to SEP. Second, this study did not use sampling weights for multilevel analyses, which implies that the present findings are not representative of the study population. Third, the data used were relatively old (2009). However, this is the most comprehensive data on use of preventive services available across Europe. Most importantly, there is no evidence of changes in inequalities in use of services in Europe over recent times [38]. Fourth, the survey did not include information on private medical and/or dental insurance at the individual level, which could have had an impact on the findings. Hence, healthcare system as a country-level measure was included in the analysis. Fifth, there was no information on health status or medical/dental history in the survey which could have affected the results. However, we argue that the nature of preventive services such as routine medical screening (for breast cancer or blood pressure) or dental check-ups should be independent from an existing condition, or family history. Finally, there is always the possibility of reporting bias in surveys, particularly in relation to SEP indicators and use of preventive services.

## 5. Conclusions

Clear social gradients in use of preventive dental and medical services among European adults were observed. Only dental check-ups maintained significant gradients across all objective and subjective socioeconomic indicators, although cancer screening showed inequality in education and subjective social status, and cardiovascular disease screening did in difficulty in paying bills. Targeting socioeconomic inequality would improve the preventive attendance of medical/dental screenings.

## Figures and Tables

**Table 1:** Characteristics of the study sample and proportion of people using dental and medical preventive services in the past 12 months, by covariates (*n* = 23,464).

Explanatory Variables	All Sample		Dental Check-Ups	Cancer Screening	CVD Screening
	*n*	%			
**Gender**					
Male	10,254	47.9	61.1	22.3	67.6
Female	13,210	52.1	64.9	25.7	71.5
*p*-value ^a^			<0.001	<0.001	<0.001
**Age groups** ^b^					
20–29 years	3185	16.4	62.7	7.3	49.7
30–39 years	4142	19.1	65.7	12.7	54.8
40–49 years	4153	18.8	67.1	21.2	66.6
50–59 years	4208	16.7	65.1	34.8	78.4
60–69 years	4016	14.9	63.4	39.7	86.0
70+ years	3760	14.1	51.9	33.5	89.5
*p*-value ^a^			<0.001	<0.001	<0.001
**Area of residence**					
Rural area or village	8539	35	59.7	23.6	70.4
Small or middle-sized town	8224	40.1	65.0	25.7	71.0
Large town	6701	24.8	64.6	22.0	66.3
*p*-value ^a^			<0.001	<0.001	<0.001
**Marital status**					
Married	15,561	66.7	65.3	26.1	70.6
Single	7903	33.3	58.6	19.9	67.7
*p*-value ^a^			<0.001	<0.001	<0.001
**Healthcare system**					
Nordic	2818	4.16	73.1	21.2	63.1
Bismarckian	5393	35.8	70.5	35.1	74.1
Beveridgean	1974	12.8	66.5	19.0	59.9
Southern	4465	26.6	59.0	18.9	70.1
Eastern	8814	20.5	51.2	15.3	68.6
*p*-value ^a^			<0.001	<0.001	<0.001

CVD: cardiovascular disease. ^a^ Chi-squared test was used for comparisons. ^b^ These categories were used for presentation purposes only.

**Table 2:** Crude socioeconomic inequalities in use of dental and medical preventive services among adults aged 20+ years old in European Union countries (*n* = 23,464).

Socioeconomic Measures	All Sample		Dental Check-Ups	Cancer Screening	CVD Screening
	*n* ^a^	%			
**Education**					
20 years or more	7609	30.2	70.0	22.3	65.6
16–19 years	10,689	45.2	65.2	23.0	66.9
Below 15 years	5166	24.5	50.7	28.2	79.6
*p*-value for trend ^b^			<0.001	<0.001	<0.001
**Difficulty of paying bills**					
Almost never/Never	15,055	66.0	67.3	26.5	71.3
From time to time	6241	26.2	57.8	20.2	67.4
Most of the time	2168	7.7	44.8	16.7	63.3
*p*-value for trend ^b^			<0.001	<0.001	<0.001
**Subjective social status**					
Highest	6774	27.1	73.2	25.1	67.4
Second highest	5008	24.3	66.3	24.9	69.1
Second lowest	6816	30.0	59.9	24.8	72.2
Lowest	4866	18.5	49.3	20.4	69.5
*p*-value for trend ^b^			<0.001	<0.001	0.008

CVD: cardiovascular disease. ^a^ Counts are unweighted. ^b^ *p*-values for trends were derived from crude logistic regression models where the SEP indicator was fitted as a continuous variable.

**Table 3:** Socioeconomic inequalities in use of dental and medical preventive services among adults aged 20+ years old in European Union countries.

Fixed Effects	Dental Check-Ups	Cancer Screening	CVD Screening
	OR ^a^ (95% CI)	OR ^a^ (95% CI)	OR ^a^ (95% CI)
**Individual-level variables (*n* = 23,464)**			
**Education (reference: 20 years or more)**			
16–19 years	0.79 (0.73–0.84) ***	0.87 (0.81–0.95) **	0.88 (0.82–0.95) **
Below 15 years	0.51 (0.46–0.55) ***	0.68 (0.61–0.75) ***	0.93 (0.84–1.03)
**Subjective social status (reference: highest)**			
Second highest	0.86 (0.79–0.94) **	0.90 (0.82–0.98) *	0.94 (0.86–1.03)
Second lowest	0.75 (0.70–0.82) ***	0.82 (0.75–0.90) ***	0.94 (0.86–1.03)
Lowest	0.63 (0.57–0.69) ***	0.71 (0.63–0.79) ***	0.78 (0.71–0.87) ***
**Difficulty in paying bills (reference: never)**			
From time to time	0.82 (0.76–0.88) ***	0.99 (0.91–1.07)	0.87 (0.81–0.94) ***
Most of the time	0.64 (0.58–0.72) ***	0.97 (0.85–1.11)	0.81 (0.73–0.91) ***
**Gender (reference: male)**			
Female	1.41 (1.33–1.49) ***	1.23 (1.15–1.31) ***	1.31 (1.23–1.39) ***
Age in years	0.99 (0.99–0.99) ***	1.03 (1.03–1.03) ***	1.05 (1.04–1.05) ***
**Marital status (reference: married individuals)**			
Single	0.73 (0.68–0.77) ***	0.72 (0.67–0.78) ***	0.90 (0.84–0.96) **
**Area of residence (reference: rural area or village)**			
Small/middle sized town	1.05 (0.98–1.12)	1.02 (0.95–1.10)	1.14 (1.05–1.22) **
Large town	1.12 (1.04–1.20) **	1.12 (1.03–1.21) **	1.10 (1.02–1.19) *
**Country-level variable (*n* = 27)**			
**Oral healthcare system (reference: Nordic)**			
Bismarckian	1.18 (0.65–2.16)	2.10 (1.31–3.38) **	1.73 (0.94–3.18)
Beveridgean	0.74 (0.36–1.51)	1.01 (0.57–1.78)	1.15 (0.56–2.38)
Southern	0.60 (0.32–1.12)	1.11 (0.68–1.82)	2.02 (1.08–3.78) *
Eastern	0.52 (0.29–0.93) *	0.86 (0.55–1.36)	2.03 (1.14–3.64) *
**Random effects**			
Country-level variance (SE)	0.19 (0.05)	0.12 (0.03)	0.20 (0.05)
Variance partition (%)	5.5%	3.5%	5.7%
Median Odds Ratios (95% CI)	1.52 (1.42–1.61)	1.39 (1.33–1.44)	1.53 (1.43–1.63)

^a^ Two-level binary logistic regression was fitted (participants nested within countries). Odds ratios (OR) were therefore reported, which were adjusted for all the variables in the table. * *p* < 0.05; ** *p* < 0.01; *** *p* < 0.001
